# DISTINCT PROFILES OF SEDENTARY BEHAVIORS IN OLDEST OLD ADULTS

**DOI:** 10.1093/geroni/igac059.1300

**Published:** 2022-12-20

**Authors:** Katelyn Webster, Weijiao Zhou

**Affiliations:** Indiana University, Indianapolis, Indiana, United States; University of Michigan, Ann Arbor, Michigan, United States

## Abstract

Sedentary behavior (SB) is a significant health risk. Emerging research suggests that mentally active SB (such as computer use and reading) were associated with better health than mentally passive SB (such as watching TV). However, this has not been examined among the oldest old (age ≥80). The aims of this study were to (1) identify distinct profiles of oldest old adults based on six domains of SB (watching TV, using a computer/tablet, talking to friends or family members, doing hobby or other activities, transportation, and resting/napping); and (2) compare health-related outcomes across identified profiles, using the National Health and Aging Trends Study (NHATS) dataset. Latent profile analysis was used to identify distinct profiles of SB. Design-based linear and logistic regressions were used to examine associations between different profiles and health outcomes, accounting for sociodemographic characteristics. We identified four profiles and named them based on total sedentary time (ST) and passive/active pattern (n=852, “Low ST”, “High ST-passive”, “Medium ST-TV”, “High total ST-mentally active”). Compared to the “High ST-passive” group, “Low ST” group was associated with fewer difficulties with activities of daily living, fewer problems limiting activities and higher cognitive function; “High ST-mentally active” group was associated with the above outcomes, as well as lower anxiety and depression. This study, with a national representative sample of the oldest old population, suggests that both total ST and SB pattern matter when evaluating health outcomes of being sedentary. Interventions should encourage oldest old adults to reduce ST and especially target mentally passive ST.

